# How to play the game of medical education: Sociocultural analyses of educational difficulties among medical residents

**DOI:** 10.15694/mep.2020.000125.1

**Published:** 2020-06-18

**Authors:** Mette K. Christensen, Johanne K. Sørensen, Rune D. Jensen, Signe G. Brøndt, Karen Norberg, Lotte D. O'Neill, Lene S. Mortensen, Peder Charles

**Affiliations:** 1 Aarhus University; 2 Northern Postgraduate Medical Training Region Secretariat; 3 University of Southern Denmark; 4 Department of Internal Medicine

**Keywords:** Residents in difficulty, Residency training, Struggling trainees, Pierre Bourdieu, Clinical learning environment, Learning climate, Organisational culture, Workplace learning, Coping strategies, Professional identity.

## Abstract

This article was migrated. The article was marked as recommended.

Medical residents in difficulty struggle to comply with educational requirements. They pose a liability to patient safety and they have problems to adapt to the professional role of a doctor. Consequently, being a resident in difficulty may cause identity crisis and have the potential to disrupt the resident’s professional identity as a doctor. Only few studies explore the tipping point between becoming a resident in difficulty or not, and these studies rarely reflect the surrounding sociocultural aspects of the residents’ difficulties such as organisational culture in the workplace. This article explores how medical residency training culture influence on residents’ risk of ending in difficulty. Our study was based on six focus-group interviews with residents (n=28) and in-depth interviews with residents in difficulty (n=10). The interpretation of data employed sociologist Pierre Bourdieu’s theoretical framework around dispositions. Across the data, we identified four themes: Conflicting games in the field of medical education, altruism, organisational hierarchy, and coping with stress. We found a (mis)match between legitimate rules in the field of medicine and the residents’ dispositions to appreciate those rules. These results can inform clinical supervisors and consultants in their decisions for supporting residents in difficulty and increasing educational achievement among struggling residents.

## Introduction

Residents in difficulty are resource demanding for healthcare institutions ( Roberts *et al*., 2012; Williams, Roberts, Schwind, and Dunnington, 2009). In addition, ongoing educational difficulties may jeopardise the resident’s professional career ( Ringsted, 2014), and would seem to cause significant problems to the resident’s professional identity and self-image ( Good, 1994; Kaiser, 2002). Consequently, being a resident in difficulty may cause identity crisis, insecurity and anxiety, and may have the potential to disrupt the resident’s professional identity as a doctor ( Cruess, Cruess, and Steinert, 2016). International studies show that 3-10 % of doctors in postgraduate specialist training struggle to comply with educational requirements ( Christensen *et al*., 2016; Tabby, Majeed, and Schwartzman, 2011; Zbieranowski, Takahashi, Verma, and Spadafora, 2013). Generally, the issues concerning residents in difficulty are addressed in retrospective quantitative, descriptive studies of the prevalence and behaviours of residents facing difficulty ( Adams, Emmons, and Romm, 2008; Brenner, Mathai, Jain, and Mohl, 2010; Dupras, Edson, Halvorsen, Hopkins, and McDonald, 2012; Yao and Wright, 2000; Zbieranowski *et al*., 2013). The aforementioned studies have contributed to knowledge of the number of residents in difficulty in different specialties, the behaviours of individual residents in difficulty, the management and remediation of residents in difficulty, and personality traits that may identify a resident at early stages of difficulty. Yet, it could be argued that these studies did not reflect the surrounding sociocultural aspects such as organisational culture in terms of learning climate, working hours, working climate, social structure, and hierarchy in the workplace. Organisational culture has an immense impact on residents’ education ( de Cossart and Fish, 2005). However, Christensen *et al*. (2016) found that only few studies explore sociocultural aspects of postgraduate medical education that would help to explain why some residents end up in difficulties, and the tipping point between becoming and not becoming a resident in difficulty is still a puzzle. Therefore, in this study, we aim to expand the field of research on residents in difficulty by exploring residents’ experiences of medical residency training culture and its influence on residents’ risk of ending up in difficulty. Thus, we based this qualitative study on reflexive sociological analysis of residents’ stories about difficulties in postgraduate medical education. More specifically, we introduce sociologist Pierre Bourdieu’s concepts *doxa*, *habitus* and *illusio* ( Bourdieu, 1992; Bourdieu, 1998; Bourdieu and Wacquant, 1992) as a theoretical framework for understanding the organisational culture and educational difficulties in the field of medicine. These concepts provide a useful lens for investigating norms, practices and ‘drivers’ along with other sociocultural aspects of residents’ socialisation and learning processes.

### Theoretical framework

The concepts of Bourdieu are closely linked to his theory of power relations and his attempt to understand how power, including the power of social norms, works without explicit coercion ( Grenfell, 2014). Bourdieu understood the social world as a multidimensional social space consisting of multiple fields in which interests, conflict and competition appear simultaneously ( Bourdieu and Wacquant, 1992), thus his understanding of society can be illustrated by metaphors of ‘a game’ with rules and a playing field. A field is constituted by *doxa*, that is explicit as well as tacit rules, norms and beliefs that inform the hared habitus of those operating within the field. In order to occupy certain positions in the field, agents (such as medical residents) must learn to play the game and know what is required to produce adherence of the field’s norms and practices in order to appreciate and obtain legitimate forms of knowledge, understandings and interests - also termed *illusio.* The determining link between the objective structures in the field and the way in which the individual agent plays the game is termed *habitus.* In this study, we do not regard medical education as a field into itself, but as a particular ‘game’ in the overarching professional field of medicine in which postgraduate medical education is just one of many practices. One can argue that medical education is parasitic on medicine: it eats into and is feeded by the professional field of medicine and it interests with other subfields of practice, e.g. various specialties. Below, we expand on the key concepts *doxa, habitus* and *illusion* in relation to the topic of this study.

*Doxa* underpins both the orthodox and unorthodox points of view in a field and it is often expressed in daily practices and sayable as well as unsayable ways of doing things: “ *Doxa* is [...] the preverbal taking-for-granted of the world that flows from practical sense” ( Bourdieu, 1992, p. 68). It relates to deep structures in a field, that is often unconscious, unquestionable norms and presuppositions of ‘the game’, and is thus necessary for maintaining order in the social fields - an order that provides an underlying basis for what to do and not to do in a field ( Bourdieu, 1998). The development of *doxa* and its legitimation of norms, practices and positions in the field are reinforce by the dominant agents through their exercise of *symbolic violence* (by imposing meanings and ways of thinking) over other agents in the field. According to Bourdieu (1998), symbolic violence is exercised upon a social agent with his or her accept and participation, because it is “based on “collective expectations” or socially inculcated beliefs” (p. 103). It works with the tacit acceptance of both those who use it and those who are subject to it, because both parts are interested in occupying positions in the field.

*Habitus* is a concept that denotes the individual agent’s mental and bodily dispositions, i.e. incorporated schemata of perception, appreciation and action. Habitus is produced and reproduced by interactions between an agent and a field, and it is the dynamic internalisation of skills, which operate according to the norms and logic of the field and its *doxa.* According to Bourdieu (1992), *habitus* is always already oriented towards practical function, because it is a system of “durable, transposable dispositions, [...] principles which generate and organize practices and representations that can be objectively adapted to their outcomes without presupposing a conscious aiming at ends” (p. 53). Consequently, since large parts of *habitus* are affected by social and cultural norms, *habitus* is field relative, parts of which are shared with some, e.g. colleagues, and not others. In fields such as medicine a certain degree of shared medical habitus is required to maintain order, and thus, “in a Bourdieuan perspective, the implicit purpose of medical education is the (re)production of the medical habitus” ( Emmerich, 2013, p. 30).

*Illusio* describes an agent’s sense of the ‘game’ and the capability to take seriously both the forms of capital in a particular field and the rules of acquiring it. This capacity may result in an experience of being caught up by the game and finding it interesting and fulfilling, and a game worth playing ( Bourdieu, 1998). Bourdieu probably would not use psychological words such as ‘motivation’ or ‘dedication’, but they help to explain *illusio* as a matter of the match between *doxa* and *habitus.* In the present study, *illusio* is a pivotal concept in understanding the relationship between the *habitus* of residents and the *doxa* in the field of medicine. It explains how a resident’s interest and immersion in this particular field of practice requires that he or she becomes dedicated to buying into and internalising the underlying logics of this particular practice and stays caught up in and by the game.

A reason for there being a tipping point between becoming and not becoming a resident in difficulty could be the ways in which *illusio* unfolds in the match or mismatch between the *habitus* of the residents and the *doxa* in the field of medicine. Thus, the present study addresses the question:

• How does the organisational culture of residency training (understood through the concepts of *doxa, illusio* and *habitus*) influence on residents’ risk of ending up in difficulty?

The purpose of the study was twofold: 1) to gain insight into residents’ subjective experiences of socialisation into the medical field including the ‘game’ of medical education, and 2) to compare the experiences of a group of residents in difficulty and a group of residents with no recorded educational difficulties - those who followed what we may call straightforward educational courses. For this purpose, we conducted a comparative study to explore differences and similarities concerning the challenges met by these two different groups of residents. Our goal was to identify structures in the organisational culture of residency training that can inform decisions for supporting residents in difficulty and thereby create knowledge that could be used to decrease the experienced difficulties and risk of educational delay. We applied the conceptual tools of Bourdieu in combination with medical anthropological perspectives ( Good, 1994) on doctors’ professional socialisation to qualify our analysis of profession-specific educational dilemmas.

## Methods

Applying a qualitative methodology, our study relates to the social constructionist understanding of storytelling ( Mattingly, 1998) as being integral to the comparative analysis of residents’ perspectives, incorporating personal experiences with difficulties in residency training. In order to achieve a wide variety of perspectives on our research question, we based this empirical study on in-depth interviews ( Kvale and Brinkmann, 2009) with residents in difficulty and focus group interviews ( Stalmeijer, McNaughton, and Van Mook, 2014) with residents in straightforward educational courses.

### Data collection and participants

We recruited participants through gatekeepers in the field of medical education in Denmark. A gatekeeper is a person who stands between the data collector and a potential participant ( Shenton and Hayter, 2004). Each gatekeeper received the same information letter about the research project. The letter included an email to the residents in which we invited the resident to participate in interviews and to contact a project assistant (third author) for further information. In this way, the gatekeeper would not know if the resident participated, and all residents were guaranteed anonymity.

The participants in the in-depth interviews were residents in difficulty, that is, these residents had been reported as having critical difficulties and for different reasons had extended employment periods to attempt to achieve the required competences in their specialist training. The gatekeepers in the in-depth interviews were postgraduate clinical associate professors (PCAP) entrusted with the role of educational mediators within their respective specialties in cases where residents were in difficulty. We encouraged the 53 PCAPs in two of the five healthcare regions in Denmark to e-mail the information about the research project to 96 resident(s) in difficulty. Thirteen of the 96 residents contacted the project assistant, 10 of whom volunteered and took part in in-depth interviews. The participants (8 females/2 males) came from six different medical and surgical specialties.

The participants in the focus group interviews were residents in straightforward educational courses who progressed as intended in residency training. We recruited the participants through two gatekeepers: 1) heads of departments in one hospital, and 2) teachers of mandatory seminars for residents in the two healthcare regions. We encouraged the gatekeepers to distribute the information about the research project to the departments’ residents and in the seminars. Twenty-eight residents (15 females/13 males from a range of surgical and medical specialties) volunteered and were assembled in five groups of 5-6 participants with both female and male residents in each group.

### Interviews

We conducted ten in-depth interviews (lasting between 45 and 100 minutes per interview, averaging 77 minutes) and five focus group interviews (lasting between 54 and 108 minutes per interview, averaging 78 minutes). In order to generate comparative interview data, we used comparable semi-structured interview guides ( Kvale and Brinkmann, 2009) in both the in-depth and focus group interviews. The interview guides comprised open-ended questions that were thematically structured with respect to five themes identified in a previous study of residents in difficulty ( O’Neill *et al*., 2014; Christensen *et al*., 2016): Theme 1: Lack of mutual expectation and role clarification between resident and workplace; Theme 2: Lack of time to complete tasks; Theme 3: Difficulty in maintaining work-life balance; Theme 4: Experience of insecurity and anxiety; and Theme 5: Symptoms of stress. In addition to this, we invited the focus groups to reflect on the strategies or methods they used to avoid difficulties in order to collect useful experience-based data about coping strategies.

### Analysis

All interviews were transcribed verbatim. We chose an abductive approach to the analysis ( Tavory and Timmermans, 2014) which we conducted in three steps. Firstly, we categorised the transcribed interviews into the five themes of the interview guide and coded quotes pertaining to the themes. For this coding procedure, we used the research software Nvivo10. This first step served as an immediate comparative analysis of the two groups of residents. As expected, this procedure revealed that large amounts of the data did not correspond with the predefined themes. According to Tavory and Timmermans (2014), “abduction occurs when we encounter observations that do not neatly fit existing theories and we find ourselves speculating about what the data plausibly could be a case of [...] Abduction produces a new hypothesis for which we then need to gather more observations” (p. 5). Hence, the achieved knowledge was used to pose new analytical questions. Secondly, we revisited the transcribed in-depth interviews, summarised key stories in concentrated narratives of proximately five pages each describing the participants’ experiences and reasons for their challenges. Thirdly, we conducted a theoretical reading ( Kvale and Brinkmann, 2009) of the narratives generated in the second step of the analysis along with the complete interviews. In this theoretical reading, we applied the theoretical framework of Bourdieu to interpret the data. Analytical mindmaps were drawn to visualise analytical interrelations between our data and the theoretical concepts. In this process, a medical anthropological perspective ( Good, 1994) on doctors’ professional socialisation were included and the analysis was directed at contradictions and social conflicts in the organisational culture. This procedure produced four clusters of sociocultural aspects of residency training (the four themes in the results section) that may explain the tipping point between becoming or not becoming a resident in difficulty. A few selected and illustrative quotes and narratives from the interviews are presented in Table 1- 5 in the results section.

### Ethical considerations

The study was ethically approved by the Danish Data Protection Agency (J. No. 2013-41-1794). Approval from the Central Denmark Regional Committees on Biomedical Research was not required. The American Anthropological Association’s code of ethics was followed ( American Anthropological Association, 2012). In particular, we chose not to specify which hospitals, wards and medical specialties we included in the study to maintain the participants’ anonymity and enable them to speak freely. All participants in this study were informed and accepted (both in writing and orally) before the interviews commenced that data would be treated confidentially and anonymously and used in a publication. Consequently, participants’, persons’ and places’ real names have been removed in order to preserve anonymity and ensure confidentiality.

## Results/Analysis

We identified four themes characterising the organisational culture of residency training which influence residents’ risk of ending up in difficulty: 1) Conflicting expectations - ‘the game of education’ and ‘the game of production’, 2) From altruism to pragmatism, 3) The organisational hierarchy and the residents, and 4) Coping with stress and system pressure: sharing expectations, adjusting standards or escape strategy. Below, we present each of the four themes and include selected quotes from the data.

### Conflicting expectations - ‘the game of education’ and ‘the game of production’

As medical students, the residents had gained considerable theoretical knowledge and little practical experience. However, working in clinical departments immediately after medical school meant that they were confronted with real patients with complex pathologies and were supposed to learn diagnostic processes and treatment in real life settings with limited economic resources and a high workload due to hospital waiting lists needing to be cut. According to the participants in our study, most clinical departments claimed to have an interest in high-quality education, and most residents expected an employment with focus on educational outcomes. However, the shortage of economic and professional resources compromised both the everyday clinical management and treatment of patients - ‘the game of production’ - and the educational endeavours predicated on it - ‘the game of education’ - despite the continuing demand for well-educated physicians. These contradictory expectations were reflected in all of the interviews with both groups of residents (see Table 1).

**Table 1.** Quotes about conflicting expectations

Quote 1	“From day one, the department expected me to handle patients, which I didn’t feel I could. I had expected to get educated, and they expected that I was educated” (Resident in difficulty 1).
Quote 2	“It is expected that you pitch in. If you do not, it is impossible for the department [and me] to catch up with the assignments, and you can be sure that you will not be employed after your residency period” (Resident in difficulty 2).
Quote 3	“I felt the expectations were high. They (the department) expected that I had learned more than I actually had during my residency. It was almost impossible to satisfy all their demands” (Focus group 2).
Quote 4	“From day one, you get thrown right into it. I had some horrible shifts, working non-stop. I felt poorly equipped for the expected amount of responsibility. I literally had to run during my ward rounds to catch up with my schedule [...and] I had mixed feelings of bitterness and professional embarrassment” [...]“Clarification of roles is not a part of Danish hospitals. You just try to fit in and get comfortable in the hierarchical structure and social constellations. You learn it along the way, but it is very tacit and taken-for-granted” (Resident in difficulty 3).
Quote 5	“It’s damned hard for me to accept that I make mistakes. Doctors [like me and other residents] are often clever people. They have studied for years; they have good grades and so on. Suddenly, we have to accept doing our jobs just adequately and not perfectly. Moreover, we make mistakes on almost every shift” (Focus group 4).

On the one hand, the residents worked as doctors in highly specialised departments with very tight schedules where all employees needed to work as skilled professionals. On the other hand, they were learners and expected educational guidance and time for training along the way. Some participants told about understaffed clinical departments, which placed even greater pressure on all of the employees in the clinical departments to focus on ‘the game of production’, leaving various educational problems unaddressed. Quote 4 in Table 1 and many more in our interviews with both groups of residents echo a typical condition of many of the residents’ workplaces that reflects an underlying doxastic pragmatism to medicine that means the game of production is always prioritised over any other games. The rhetoric of prioritising education does not conflict with the underlying doxa; although, in practice, education is clearly sacrificed on the altar of pragmatism. From our data, it became apparent that the antithesis between the rhetoric and the practice in the clinical departments’ *doxa* caused experiences of disappointment and powerlessness in both groups of residents in this study. Some participants (for example quote 4 and 5 in Table 1) shared their frustration about the unspoken and taken-for-granted role of residents and the clash between being a medical student and being a medical professional.

From our interviews, it seemed that the *doxa* of medicine feature an unspoken expectation that residents learn by doing while playing the game of production. At the same time, the residents felt that they intuitively ought to know how to act in the clinical departments’ hierarchies, while the different clinical departments each seemed to have their own field-relative departmental habitus. In Denmark, the residency programme encompasses 2-4 different employments of 6-36 months. Variability within departmental *habitus* made it difficult for residents to learn how to act in the various departmental settings, and decode and incorporate the *illusio* of specific workplaces. These conflicting expectations - ‘the game of education’ and ‘the game of production’ - indicated that neoliberal agendas in education ( Connell, 2013) and in the healthcare sector ( McGregor, 2001) associated with cost-cutting efficiency measures while simultaneously cutting down the human resources subsequently joined with the *doxa* of medicine to produce the results above. As shown in our interviews, this affected both groups of residents negatively as they experienced systems-related barriers in getting their educational training and thereby in achieving the competences and self-efficacy they needed. Literally, they were caught in between the two games being played, that of production (clinical practice) and of education (residency training). According to our data, conditions are such that the two games pull in different directions although they are submitted to the same neoliberal agenda, and production has priority over education. - and this appeared to be rather stressful for both groups of residents.In the following, we will focus on how the individual residents and their vision of clinical work was fulfilled or negatively affected by the double bind between the *long-term* perspective of the education game and the *short-term* perspective of the production game.

### From altruism to pragmatism

Both groups of residents in this study told that in general they chose to study medicine for altruistic reasons as they felt committed to helping others, but the reality of the clinical departments gradually made them adopt a more pragmatic approach to their jobs (see Table 2). Inherent in the transition from altruism to pragmatism ( Good, 1994; Harris, 2018) was the conflict between the games of education and production. In other words, there was an imminent contradiction within the *illusio* of the organisational culture. These tensions affected the residents in the guise of time pressure and experienced lack of educational support. Altogether, this clashed with their professional altruistic ambitions of perfect examination and diagnostication. With insufficient time for looking things up or practising, residents became disillusioned or even unhappy about their demanding work conditions and experienced low priority to educational training, and feared the consequences this could have on their patients in terms of medical errors.

**Table 2.** Quotes about altruism and pragmatism

Quote 1	“I would be a much more happy resident if I had more time to spend on my tasks. What makes me feel miserable is actually that being a doctor is the best thing in the world. It is so frustrating not having the time to acquaint myself fully with the patient or the task before I need to act. Instead, I need to ask somebody, so I never learn” (Focus group 4).
Quote 2	“We are understaffed. I am totally exhausted for 3 days after a 24-hour shift. Sometimes, there is no time to go to the lavatory. During my first employment, we residents were told to admit even more patients. But we were too slow to take care of all those patients. It was all about the hospital management and economy” (Resident in difficulty 4).
Quote 3	“During stressful periods, there is no time to think twice when you see a patient. You just need to conduct the consultation quickly and decide on the choice of treatment right away. Of course, it can be stressful, but you need to learn how to act under time pressure” (Focus group 3).
Quote 4	“It is so important for a resident to be able to work under time pressure. If you cannot handle the lack of time during patient consultations, it is very, very difficult to be a doctor” (Focus group 2).

Another aspect relating to their perception of considerable work pressure and limited time in clinical departments was that it left residents with a feeling of dealing superficially with patient examinations and diagnosis, which filled some of them with feelings of insufficiency and even anxiety that they might have overseen something important. Many of the residents expressed worries and frustrations about making mistakes due to the time pressure in clinical departments (see quote 3 and 4 in Table 2). In addition, in our interview data, doctors were labelled as “clever people” with “good grades” and people with high ambitions and expectations of themselves, and residents in our study expressed frustration saying, for example: “we have to accept doing our jobs just adequately and not perfectly” and “it was almost impossible to satisfy all their demands”. It seemed that time pressure and a gradual transition from altruism to pragmatism was an indisputable part of their work and education in clinical departments. This particularly affected newcomers and residents who might need a little more time to learn about specific work procedures, routines and decision-making. Consequently, time pressure as a *doxic* factor in the field of medicine disrupted many of the residents’ *illusio* about being a good doctor, that is, an altruistic doctor. Especially residents in difficulty expressed that without this particular *illusio*, it was almost impossible (or even hopeless) to play any of the two games well.

### The organisational hierarchy and the residents

A thorough understanding of the *illusio* of a workplace is a precondition for playing the game well and for succeeding as a resident. Understanding how to act within both the formal and informal professional hierarchy of a department with its various assumptions, values and traditions was very important for residents. Some residents stated that the department expected the newly employed resident to intuitively know the hierarchy of the department. The quotes in Table 3 underline how important it was to be aware of the organisational structures and the rules of the game within them.

**Table 3.** Quotes about the organisational hierarchy and the residents

Quote 1	“In some departments, it is more clearly articulated than in others. The hierarchy can be compared to a military system: if you can act in a military system, I think it is easier to act in a hospital” (Focus group 5)
Quote 2	“It (the hierarchical structure) was very uncomfortable, but it did not come as a surprise. Actually, I felt a bit surprised about how uncomfortable it was, but again that is just how it is” (Focus group 1)
Quote 3	“My supervisor expected me to be a copy of her, so when I engaged in the political part of our profession, it clashed with her comprehension of how a real doctor should act” (Resident in difficulty 6)

However, a ‘feel for the game’ (Pierre Bourdieu, 1998), i.e. a match between *habitus* and *field*, is not enough to succeed as a resident. External factors that relate indirectly to the organisational hierarchy, such as the dominant position of a chief physician, could also influence individual residents and their professional development. This is illustrated in the case of Anna (pseudonym, resident in difficulty 5), a young resident in her specialisation programme. The case is an extreme but real case that illustrates the harsh conditions a young doctor may endure when she meets the *symbolic violence* of the organisational hierarchy. Table 4 is the abbreviated narrative, where Anna explained how she felt after a chief surgeon made use of his superior position on her first day attending the morning conference.

**Table 4.** The case of Anna

Abbreviated narrative	*“It was my first day at this department. We were at the morning conference. One of the chief surgeons, who was appointed to be my supervisor, turns to me and says in a harsh manner: “You are a small, foreign woman, what are you doing in a surgical department?”. I was really upset by this, and a few minutes later, he says to the rest of those attending the morning conference: “She is supposed to specialise with us, but I do not think she deserves it”. No one said anything. Afterwards, only a few colleagues commented on it and told me just to accept his behaviour - but I simply could not. Later that day, he asked me to come to his office, where he told me: “You’ve got your residency here, but I do not agree. I will do everything I can to ensure that you do not get your specialisation. Get out.” This day affected me a lot. After my shift, I walked by the lake. I was thinking that I might as well take my own life. I was so frustrated. I could not bear telling my husband the bad news. But then again, I wanted to be there for him and for my child” (Anna).*

Anna regarded herself an ambitious and competent physician. She had been offered positions elsewhere, but had decided on surgery. However, as shown in the above quotation, rejection was based on her bodily attributes, discrimination and prejudice, and not her medical proficiency as a future surgeon. Within the department, there appeared to be a *doxa* that colleagues did not contradict this particular chief surgeon. The order of the field was hierarchical and the norm was that one does not challenge the hierarchy of medicine, something that this surgeon had clearly reinforced in relation to himself. Accordingly, colleagues observed the personal insult at the morning conference in silence. The seriousness of the symbolic violence on Anna’s personal and professional identity was clearly expressed in the interview and her thoughts after that day. Her professional *illusio* was endangered and she was pushed into an existential dilemma. It appeared from this story and from similar narratives in our data that the *doxa* of the field somehow prevented colleagues at lower hierarchical levels from standing up to oppression from a superior colleague. Apparently, there was a lack of confrontational power in the department. This is also because the long-term goals (education) belong to those who occupy subordinate positions (residents) as compared to the short-term goals (production) which belong to those who occupy dominant positions (qualified medical professionals).

### Coping with stress and system pressure: sharing expectations, adjusting standards or escape strategy?

In our study, both groups of residents were caught between the coexisting, complimentary and contradictory games of production and education. At the same time, those residents are being called upon to act altruistically and to sacrifice their longer term education needs in order to meet the shorter term needs of clinical practice. These tensions along with the fact that medical errors could have serious consequences for patients contributed to an underlying theme across the interviews that related to work-related stress and system pressure. Interestingly, residents in difficulty as well as residents in straightforward educational courses presented stress as being inevitable among physicians (see Table 5).

**Table 5.** Quotes about coping with stress and system pressure

Quote 1	“Of course, you experience stress, but you need to cope with it. Crying always helps [...] You cry mostly in private, but if you’re really stressed out, it can be necessary to cry in public. I have done it once at a morning conference, and it helped. Maybe because everyone knew how tough it can get” (Focus group 1).
Quote 2	“I have often experienced events that can generate anxiety. If the event is still in my head after the shift, I always talk with my wife about it when I get home. That is always a relief” (Focus group 4).
Quote 3	“If I am really stressed, I adjust my own standards. You know, I try to be a good doctor who includes the psychological and social aspects. I also need to be a good colleague and a good dad, and stuff like that. If I am stressed, I work on my own minimum standards because my family is more important than my colleagues are. During a stressed period, I might not be the best colleague” (Focus group 1).
Quote 4	“It was so busy, I started lying about what I was doing when I had the day off. If the hospital called, I said that I was not in town, even though I was, because I seriously could not handle any more work. I just stayed inside because I was afraid my colleagues might see me” (Resident in difficulty 4).

Across the data, we identified three different stress coping strategies:

• Sharing experiences (e.g. quote 2 in Table 5)
• Adjusting standards (e.g. quote 3 in Table 5)
• Escape strategy (e.g. quote 4 in Table 5)

Residents in straightforward educational courses highlighted the importance of learning to cope with stress in order to act as a doctor, for example, by adopting coping strategies such as ‘sharing experiences’ and ‘adjusting standards’. Residents in difficulty, on the other hand, seemed to adopt an escape strategy, e.g. lying about where they were, calling in sick even if they were feeling well, not answering the phone, or going to the restroom more often while on duty. In contrast to more offensive strategies such as sharing experiences and adjusting standards this sort of defensive escape strategy was applied individually, and residents in difficulty seemed intent on dealing with stress and pressure on their own. It was not possible to determine whether an escape strategy causes or results from the transition between resident and resident in difficulty. Most likely, stress was part of the *doxa* of the professional field of medicine, that is, stress was presented as a fact of life that nothing could be done about. Thus, stress was closely related to experiences of system pressure, that is, an underlying *doxic* pressure to tolerate or even encourage extremely high workloads despite the risk of stress was incorporated in different ways by the residents.

## Discussion

Our qualitative study illustrates how a group of residents in difficulty and a group of residents in straightforward educational courses were affected by the complementary and contradictory *games* of production and education. These games overlapped in clinical settings and conflicted in a time of neoliberal educational reforms characterised by a strong politico-administrative focus on efficiency and effectiveness in the healthcare sector at large ( McGregor, 2001). In the clinical departments, there was a tight work schedule where the main aim was to cut hospital waiting lists, save patients’ lives and prevent death. Our study showed that in such a situation residents in both groups found it hard and sometimes impossible to get the educational training and supervision they needed to be the ‘good doctors’ they had dreamt of becoming. They therefore risked a disrupted *illusio.* Similar findings were found in a recent Danish study of educational challenges within clinical departments ( Skipper, Nøhr, Jacobsen, and Musaeus, 2016). The general tendency among our interviewees in both groups of residents was to describe stress as a *doxic* factor in the work culture.

To the best of our knowledge, no international studies have previously conducted a qualitative comparative study of residents in difficulty and residents in straightforward educational courses. Most international studies have focused on various documents that relate to cases of residents in difficulty, or on program directors’ perceptions of the problems they identified among residents in difficulty ( Adams *et al*., 2008; Dupras *et al*., 2012; Resnick, Mullen, Kaiser, and Morris, 2006; Roberts *et al*., 2012; Tabby *et al*., 2011). These studies appeared to identify the core of the problem as being at the level of the individual residents, paying little or no attention to organisational, sociocultural or educational challenges. A few studies were concerned with which individual residents are likely to end up in difficulty, but these studies did not find clear-cut criteria for identifying residents who ended up in difficulty ( Brenner *et al*., 2010; Paice, 2009). In this study, we showed that residency training involves a large degree of complexity, and, in Denmark at least, both educational and pedagogical principles still lag behind in the ongoing reform of postgraduate medical education ( Ringsted, 2014). In addition, neoliberal strategies in terms of reduced human resources, increased medical expenses and a growing determination to measure quality in medical education and healthcare and in terms of quantity seem to impair residency training. Recent international research shows that physicians’ mental and physical well-being is a missing quality indicator, and a key to increased treatment quality and productivity ( Wallace, Lemaire, and Ghali, 2009). In line with this, recent studies show that physicians (and their workplaces) could profit from learning efficient stress-coping strategies ( Anton *et al*., 2016; de Lasson *et al*. 2016; Koinis *et al*., 2015) in the transition from medical school to working life. Arguably, this is a further neoliberal response to a healthcare system under pressure. Instead, organisational interventions, including educational reforms, need to be better focused on addressing specific factors that cause stress (Ruotsalainen, Verbeek, Mariné, and Serra, 2014). According to the results of our study, specific factors that cause stress include sociocultural factors, in particular the ways in which the contradictory games of production and education are played and reproduced among healthcare professionals.

This study has a number of limitations. One of these is the relatively low number of participants, which may be explained by the widely held view that a resident’s difficulty is a private matter and a taboo issue. However, this limitation is deemed to be of minor importance since the findings were thoroughly discussed between the investigators, and we continued recruiting interviewees until data saturation was reached. Additionally, there could be selection bias among our self-selected interviewees who have been in difficulty. It may be that they participated out of frustrations concerning the organisational and structural challenges faced in the clinical departments. However, the aspects they identify were also reflected in the data generated from straight course residents. Both groups may also have altruistic intentions of sharing their stories in order to improve work conditions for their colleagues. It could be that there were groups of residents in difficulty we did not reach - incompetent residents, persons who were heavily challenged personally with mental problems, or the like. Nevertheless, we triangulated our difficult course interviews with straight course interviews and found many similarities in the challenges experienced by these two groups. This indicates that residents in clinical departments are faced with general structural challenges. Moreover, our comparative perspective on straight and difficult course residents appears to be rather unique within educational research concerning residents who face difficulty.

## Conclusion

Employing Bourdieu’s theoretical framework along with medical anthropological perspectives on doctors’ professional socialisation directed our focus at the interplay of social structures and general agency patterns rather than on individual residents and their personalised agency. Our inclusion of residents in difficulty and residents in straightforward educational courses in this study served to nuance the perspective of the educational and professional challenges met by residents in general. We showed how both groups of residents were affected by the contradictory games of production and education that overlap and conflict in a time of neoliberal governance and scarce resources in healthcare systems. Residents in both groups had difficulties in getting the educational training and supervision they needed in clinical departments due to scarce resources. Across the data, we identified three different stress coping strategies. Residents in straightforward educational courses had the most efficient stress-coping strategies such as sharing experiences and adjusting standards, whereas residents in difficulty applied an escape strategy that seemed to paralyse the resident. To challenge the *doxic* conception of scarce resources and stress in the health sector, we would like to pose the question: Is it possible to improve patient care and quality in treatment without improving working conditions, educational training and physician well-being?

With this study, we have made a modest contribution to the understanding of underlying sociocultural processes that influence straightforward educational courses as well as difficult ones. Both groups of residents were affected by the contradictory games of production and education that overlap and conflict in a time of neoliberal governance in healthcare systems. Thus, residents’ difficulties was a matter of *illusio*, that is, the (mis)match between legitimate explicit as well as tacit rules in the field of medicine *(doxa)* and the residents’ possibilities and dispositions *(habitus)* to appreciate those rules.

In the future, we recommend increased organisational awareness within healthcare systems of both economic and professional resources, including residents’ needs for educational training and support to reduce stress among residents and other employees. Also, we recommend that medical education focus on teaching students, residents, medical professional and supervisors a variety of efficient stress-coping strategies to better prepare young doctors to play the games of education and production and prepare the healthcare system to deal with educational difficulties. More research and subsequent intervention studies in these areas are needed to improve the quality of residents’ education and patient treatment. For this purpose, we recommend more focus on how those who occupy dominant positions in the healthcare system navigate between the games of production and education, and how those who occupy subordinate positions (residents) become critically aware of the tensions as well as overlapping interests of these two games in their everyday work culture. To answer these questions, we recommend applying sociological conceptual frameworks such as the one of Pierre Bourdieu.

## Take Home Messages

• Residents were affected by the contradictory games of production and education that overlap and conflict in a time of neoliberal governance and scarce resources in healthcare system.
• Residents in straightforward educational courses had the most efficient stress-coping strategies such as sharing experiences and adjusting standards, whereas residents in difficulty applied an escape strategy that seemed to paralyse the resident.
• We recommend that medical education focus on teaching students, residents, medical professional and supervisors a variety of efficient stress-coping strategies to better prepare young doctors to play the games of education and production and prepare the healthcare system to deal with educational difficulties.

## Notes On Contributors

**Mette K. Christensen**, PhD, is an Associate Professor at Center for Health Sciences Education, Faculty of Health, Aarhus University, Denmark. Her research interests and expertise revolve around situational, social and cultural aspects of teaching and learning in medical education.

**Johanne K. Sørensen**, PhD, is an external relations manager at School of Culture and Society, Aarhus University, Aarhus, Denmark. Her expertise is within sociological and anthopological analyses.

**Rune Dall Jensen**, PhD, is an Assistant Professor at Department of Clinical Medicine, Aarhus University, Aarhus, Denmark. He is an experienced qualitative researcher and holds a PhD in talent development in surgery.

**Signe G. Brøndt**, MD and PhD, is a general practitioner in Aarhus, Denmark. She is an experienced educator and supervisor for medical doctors, and holds a PhD in medical education.

**Karen Norberg** is administrative officer and educational consultant in Northern Postgraduate Medical Training Region Secretariat, Viborg, Denmark.

**Lotte D. O’Neill**, PhD, is an Associate Professor at SDU Centre for Teaching and Learning, University of Southern Denmark, Odense, Denmark. Her research evoles around teaching and learning in higher education and medical education.

**Lene S. Mortensen**, MD and PhD, is a consultant at Department of Internal Medicine, Randers Regional Hospital, Randers, Denmark.

**Peder Charles**, MD and PhD, is Professor Emeritus at Department of Clinical Medicine, Aarhus University, Aarhus, Denmark.
