# Supplementary material for: How to play the game of medical education: Sociocultural analyses of educational difficulties among medical residents
Source: MedEdPublish (2016). 2020 Jun 18;9:125. [Version 1] doi: 10.15694/mep.2020.000125.1 (PMC10702670; doi:10.15694/mep.2020.000125.1)
Supplement: Peer review history

# Reviewer report: Reviewer response for version 1

**DOI:** 10.21956/mep.20029.r30600

**Published:** 2020-06-26

**Authors:** Richard Hays

**Affiliations:** 1 James Cook University

This review has been migrated. The reviewer awarded 5 stars out of 5 I really enjoyed this article, which addresses a very important issue in medical education. Like other forms or professional education, medical education must dance to two different tunes simultaneously. Clinical training has elements of apprenticeship and so inevitably combines learning with the potential to affect safety, quality, efficiency and cost of care. Therefore all participants in medical education - learners, teachers and patients - are partners on this complex dancefloor. This article helps us understand how this affects learners 'in difficulty', where how to balance competing demands is less well developed than 'successful' learners. However I would like to see a similar approach used with 'successful' learners to learn how they see this complexity and manage through it. Do all regard this as a game, with rules to be followed, bent or broken? Complexity is probably unavoidable if people are to learn within health care delivery and we need to understand more about the novice to not-quite-so-novice transition, how educators can help learners through it and yet protect safety and quality. The separation of education and clinical service is hard to achieve. What I interpret from this study is that learners 'in difficulty' may benefit from 'time out' in 'safe' educational settings with slow, short periods of re-entry to clinical work with a lot of support, but then what?

Reviewer Expertise:

NA

No decision status is available

# Reviewer report: Reviewer response for version 1

**DOI:** 10.21956/mep.20029.r30601

**Published:** 2020-06-25

**Authors:** Keith Wilson

**Affiliations:** 1 Dalhousie University

This review has been migrated. The reviewer awarded 5 stars out of 5 The present study used qualitative methods to explore the ‘tipping point’ between learners that are managing well and those that are not. They presented 4 themes that emerged from their work. The first tapped into a hidden curriculum aspect that many of us involved in medicine have either overtly experienced in our own education or witnessed in the training of learners: “production has priority over education.” Other areas of discovery included transitions between altruism and pragmatism, effect of organizational hierarchy and coping with stress. Although I am not entirely clear why two different methods were used to gather data, nevertheless, the interpretation has good face validity in my opinion. However, the authors do make note of a low number of participants which could limit the generalizability of the results.There was some convergence of the experiences of both residents in difficulty and those not in difficulty. However, in their discussion, the authors delve more deeply into the differences between the two groups. They call for increased organizational awareness into the challenges noted by these residents.This paper is very well written and although some may perceive the article as very academic, the resulting themes and analysis are readily applicable to many of our learning environments. Anyone involved in the delivery of residency education will take away many helpful points from this research. The authors are to be commended on addressing a significant gap in our conversations about learners in difficulty.

Reviewer Expertise:

NA

No decision status is available

# Reviewer report: Reviewer response for version 1

**DOI:** 10.21956/mep.20029.r30598

**Published:** 2020-06-19

**Authors:** William Atiomo

**Affiliations:** 1 University of Nottingham

This review has been migrated. The reviewer awarded 4 stars out of 5 Important research question which applies to other educational contexts as well. The theoretical framework used to explore the question was innovative and interesting to read. The different interview methods in the two study groups (in-depth interview versus focus groups) was a potential methodological confounder, however, the comparable semi-structured interview guides used in both groups may have addressed this. Similar themes were also identified in both groups despite this. My only recommendation is that the key finding of the study, that “residents in difficulty, seemed to adopt an escape strategy to cope with stress, compared to residents in straightforward educational courses”, could be made more explicit in the abstract and the start of the discussion section. Good study.

Reviewer Expertise:

NA

No decision status is available

# Reviewer report: Reviewer response for version 1

**DOI:** 10.21956/mep.20029.r30599

**Published:** 2020-06-19

**Authors:** Felix Silwimba

**Affiliations:** 1 University of Lusaka

This review has been migrated. The reviewer awarded 5 stars out of 5 very informative study on the lives of subordinate residents. it is applicable across the globe.

Reviewer Expertise:

NA

No decision status is available
